# Computed Tomography Provides Improved Quantification of Trabecular Lumbar Spine Bone Loss Compared to Dual‐Energy X‐Ray Absorptiometry in Ovariectomized Sheep

**DOI:** 10.1002/jbm4.10807

**Published:** 2023-08-21

**Authors:** Katie T Bisazza, Brad B Nelson, Katie J Sikes, Lucas Nakamura, Jeremiah T Easley

**Affiliations:** ^1^ Preclinical Surgical Research Laboratory, Department of Clinical Sciences Colorado State University Fort Collins CO USA; ^2^ Orthopaedic Bioengineering Research Laboratory, Department of Mechanical Engineering Colorado State University Fort Collins CO USA

**Keywords:** BONE MINERAL DENSITY, BONE QCT, DXA, OSTEOPOROSIS, PRECLINICAL STUDIES

## Abstract

Early detection of osteoporosis using advanced imaging is imperative to the successful treatment and prevention of high morbidity fractures in aging patients. In this preclinical study, we aimed to compare dual‐energy X‐ray absorptiometry (DXA) and quantitative computed tomography (QCT) to quantify bone mineral density (BMD) changes in the sheep lumbar spine. We also aimed to determine the relationship of BMD to microarchitecture in the same animals as an estimate of imaging modality precision. Osteoporosis was induced in 10 ewes via laparoscopic ovariectomy and administration of high‐dose corticosteroids. We performed DXA and QCT imaging to measure areal BMD (aBMD) and trabecular volumetric BMD (Tb.vBMD)/cortical vBMD (Ct.vBMD), respectively, at baseline (before ovariectomy) and at 3, 6, 9, and 12 months after ovariectomy. Iliac crest bone biopsies were collected at each time point for micro‐computed tomography (microCT) analysis; bone volume fraction (BV/TV), trabecular number (Tb.N), thickness (Tb.Th), and spacing (Tb.Sp) were reported. aBMD and Tb.vBMD both decreased significantly by 3 and 6 months (*p* < 0.05) compared with baseline, whereas no changes to Ct.vBMD were observed. Combined (Tb. and Ct.) vBMD was significantly correlated with aBMD at all time points (all *p* < 0.05). Additionally, greater significant correlations were found between BV/TV and Tb.vBMD at all five time points (*R* ^2^ = 0.54, 0.57, 0.66, 0.46, and 0.56, respectively) than with aBMD values (*R* ^2^ = 0.23, 0.55, 0.41, 0.20, and 0.19, respectively). The higher correlation of microCT values with QCT than with DXA indicates that QCT provides additional detailed information regarding bone mineral density changes in preclinical settings. Because trabecular bone is susceptible to rapid density loss and structural changes during osteoporosis, QCT can capture these subtle changes more precisely than DXA in a large animal preclinical model. © 2023 The Authors. *JBMR Plus* published by Wiley Periodicals LLC on behalf of American Society for Bone and Mineral Research.

## Graphical abstract

In this preclinical study, we aimed to compare dual‐energy X‐ray absorptiometry (DXA) and quantitative computed tomography (QCT) to quantify bone mineral density (BMD) changes in the sheep lumbar spine. We observed sustained trabecular bone loss in our model using QCT, as well as greater correlations with microCT parameters, compared with DXA.

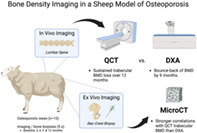

## Introduction

Osteoporosis is the most prevalent metabolic bone disease, with an estimated 200 million people affected worldwide and over 2 million incident fractures reported annually in the United States alone.^(^ ^1^, ^2^ ^)^ Osteoporotic fractures are becoming increasingly burdensome on the medical industry, resulting in exorbitant costs and high morbidity for affected patients.^(^ ^2^, ^3^, ^4^ ^)^ By 2025, the number of fracture repairs performed annually is projected to grow by 50% and surpass $25 billion in related health care costs.^(^ ^2^ ^)^ Early and accurate detection of osteoporotic bone loss is imperative for therapeutic intervention to prevent disease progression.

Clinical diagnosis of osteoporosis relies heavily on the bone mineral density (BMD) quantification of an individual patient, as fracture risk increases significantly as BMD decreases.^(^ ^5^ ^)^ BMD is most commonly measured at the femoral head or lumbar spine^(^ ^6^, ^7^ ^)^ using dual‐energy X‐ray absorptiometry (DXA). Patient BMD values are compared to those of the average healthy population using a *T*‐score to determine osteoporotic or osteopenic status,^(^ ^8^, ^9^ ^)^ wherein if the patient's *T*‐score is less than 2.5, they are diagnosed as having osteoporotic bone.^(^ ^10^ ^)^ DXA is considered the “gold standard” for clinical measurement of BMD because of its low radiation exposure and simplicity of use.^(^ ^11^ ^)^ However, because DXA quantifies bone density based on a two‐dimensional region of interest, there are limitations with its preclinical and clinical applications. Specifically, precise patient positioning and the inability to differentiate cortical and trabecular bone can lead to overerestimation of BMD,^(^ ^12^, ^13^ ^)^ thus risking underdiagnosis. Furthermore, trabecular bone is more susceptible to morphological changes during the early stages of osteoporotic bone loss in perimenopausal and aging patients.^(^ ^14^, ^15^, ^16^, ^17^, ^18^ ^)^ Other clinical factors such as obesity,^(^ ^19^ ^)^ degenerative spinal disease,^(^ ^20^ ^)^ aortic calcification,^(^ ^21^ ^)^ and osteoarthritic ostephytes,^(^ ^22^ ^)^ can also result in artificially high BMD measurements with DXA. Because of these limitations, auxiliary imaging and diagnostic strategies that provide more detailed assessments relative to DXA are needed.

Quantitative computed tomography (QCT) is emerging as an alternative screening modality to DXA by providing a volumetric quantification of BMD.^(^ ^23^, ^24^, ^25^, ^26^, ^27^ ^)^ QCT has the ability to distinguish between trabecular and cortical bone, resulting in earlier detection of low trabecular BMD and reduced overestimation issues noted with DXA.^(^ ^28^, ^29^ ^)^ Considering the superior sensitivity of QCT for early and subtle detection of trabecular BMD changes in clinical patients,^(^ ^23^ ^)^ we sought to understand if QCT provided improved quantification of bone loss relative to DXA.

Large animal preclinical models offer researchers the ability to test multiple imaging modalities and compare bone changes in the same animals over time, allowing for a more comprehensive insight into the progression of postmenopausal and age‐related bone loss. Ovariectomized sheep are commonly used in osteoporosis research because they are comparable to humans in both bone size and bone microarchitecture^(^ ^30^ ^)^ and have been shown to model decreases in bone density similar to postmenopausal women.^(^ ^31^, ^32^, ^33^ ^)^ Similarly to humans, DXA has historically been the most common imaging tool used to observe changes in lumbar spine BMD over time in the sheep osteoporosis model,^(^ ^34^, ^35^ ^)^ and the use of QCT in longitudinal research studies has been limited to high‐resolution peripheral QCT (HR‐pQCT) in the radius or tibia.^(^ ^36^, ^37^, ^38^ ^)^ QCT use has not yet been reported in the sheep spine over a long‐term study (out to 12 months post‐osteoporotic induction).

To our knowledge, QCT has not been evaluated in direct comparison with DXA to quantify BMD in the lumbar spine of sheep. Additionally, no studies have directly compared QCT, DXA, and micro‐computed tomography (microCT) changes via serial sampling over time in the same subjects—preclinically or clinically. The objectives of this study were: (i) to compare BMD measurements of the lumbar vertebrae over time in a sheep model of osteoporosis using both DXA and QCT, and (ii) to correlate QCT and DXA measurements with microCT bone microarchitecture parameters in the iliac crest of the same animals over the course of 12 months. We hypothesized that QCT volumetric BMD (vBMD) would be more sensitive to changes in trabecular bone over the course of osteoporosis progression in sheep and more closely correlate with microCT values compared with DXA aBMD.

## Materials and Methods

### Animals and experimental design

All procedures were approved by the Colorado State University Institutional Animal Care and Use Committee (protocol #2060) and were performed in an AAALAC‐accredited facility. Ten healthy skeletally mature conventionally raised Rambouillet‐cross ewes aged 4 to 6 years were enrolled in this study based on incisor presentation.^(^ ^39^ ^)^ Any animals without full incisor eruption or animals with “broken‐mouth” or heavily worn‐down teeth were excluded from enrollment. The proposed experimental sample size (10 sheep) was calculated using an a priori power analysis using longitudinal DXA BMD data from a previous study^(^ ^40^ ^)^ (G*Power version 3.1.1, Heinrich‐Heine‐Universitat Dusseldorf, Dusseldorf, Germany). This power analysis resulted in an effect size of 1.2 and a power of 90%, using a standard deviation of 0.05 for all groups. All animals were enrolled at the same time of year to avoid seasonal impacts of bone loss and fed a standard diet of alfalfa and grass hay mix with grain supplementation, as needed. Animals were cohoused in standard indoor box pen for the first 2 weeks after surgical procedures, followed by turn out to pasture for the remainder of the study.

Osteoporosis was induced in all sheep (*N* = 10) via laparoscopic bilateral ovariectomy (OVX)^(^ ^41^ ^)^ and administration of corticosteroids. Two weeks after OVX, methylprednisolone acetate (Depo‐Medrol, Zoetis, Parsippany, NJ, USA) was administered to all animals at a dose of 500 mg IM (5–7 mg/kg) every 3 weeks for a total of five doses and then reduced to half dose for three additional doses, as performed previously.^(^ ^40^, ^42^ ^)^ In vivo imaging (QCT and DXA) was performed under general anesthesia at five time points for all animals: baseline (before OVX), 3 months, 6 months, 9 months, and 12 months after OVX. Immediately after scanning at each time point, a 10 mm bone biopsy was collected from the iliac crest using an Arthrex OATS autograft system (Arthrex, Naples, FL, USA). Bone biopsy specimens were fixed in 10% neutral buffered formalin until microCT analysis. Iliac crest laterality was alternated at each subsequent biopsy time point. At the 6‐, 9‐, and 12‐month time points, the incision was placed a few centimeters away from the previous incision site and the bone was palpated for defects before collection to prevent harvesting from a previous biopsy site.

For surgical and imaging/biopsy procedures, general anesthesia was induced by injecting a combination of midazolam (0.1 mg/kg) and ketamine (3.3–5 mg/kg) intravenously into a peripheral ear venous catheter. Anesthesia was maintained using isofluorane (1.5–3%) in 100% oxygen through an endotracheal tube. Blood pressure was monitored continuously throughout the procedures either through a peripheral arterial catheter or blood pressure cuff. One day before OVX surgery and each biopsy collection procedure, transdermal fentanyl patches (150 mcg) were adhered to the forelimb for sustained release over 5 days and phenylbutazone (1 g) was administered once per day orally for 7 days for analgesic effect. Additionally, penicillin procaine G (3 million units) was administered subcutaneously once per day for 5 days for prevention of infection starting the day before each procedure.

### QCT measurements

**Fig. 1:**
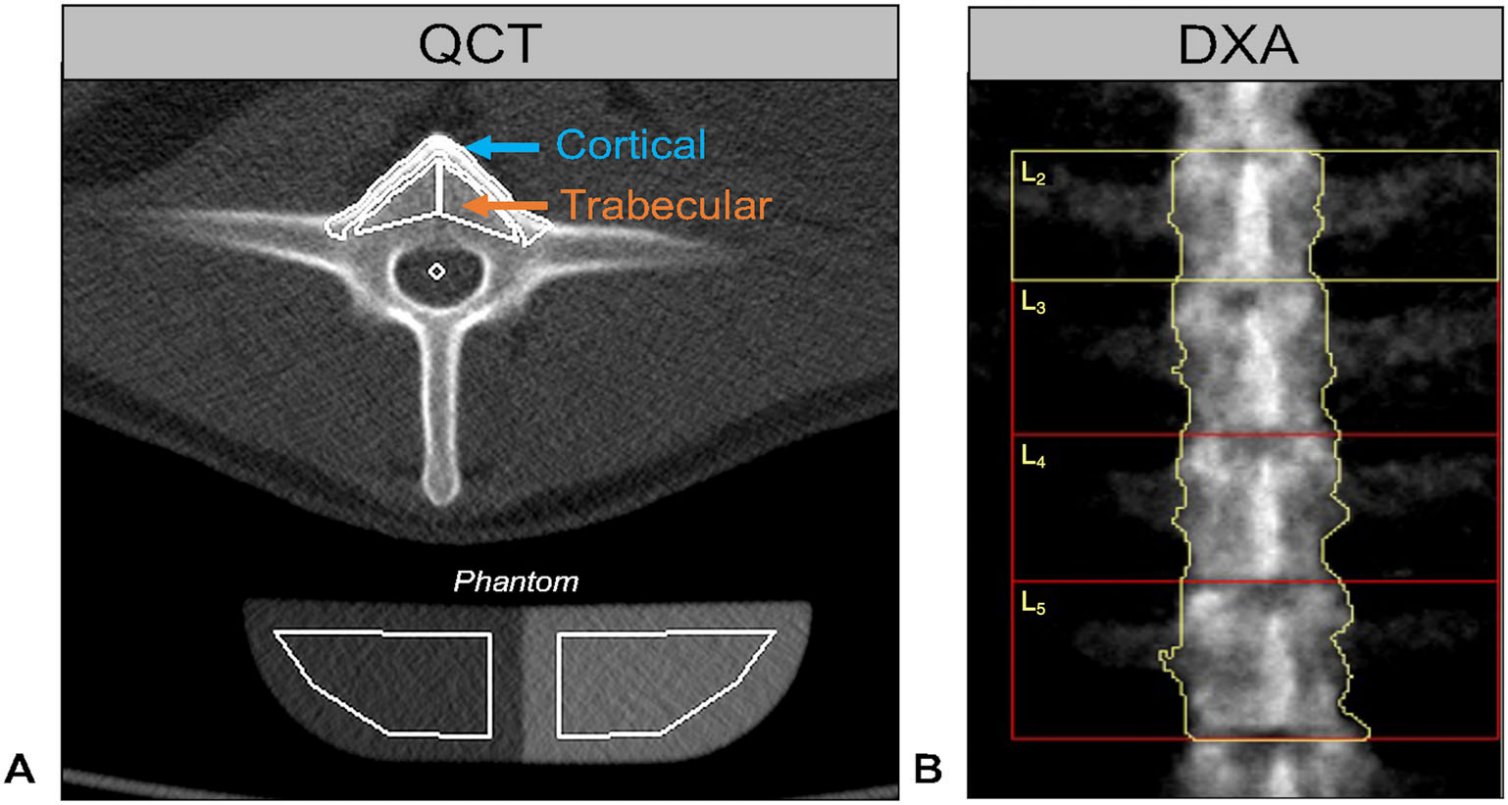
Imaging segmentation and measurement of bone mineral density (BMD) in the sheep lumbar spine. (*A*) Quantitative computed tomography (QCT) semi‐automated analysis using the syngo Osteo software. Trabecular volumetric BMD (Tb.vBMD) was determined from the inner vertebral body cancellous bone region (orange arrow) and the cortical volumetric BMD (Ct.vBMD) from the anterior cortical shell of the same vertebrae (blue arrow). Automated segmentation was verified, and manual adjustments were performed if the cortical and trabecular bone were misregistered. Hounsfield units (HU) were converted to mg Ca‐HA/cm^3^ using the water (0 mg/cm^3^, left side) and bone‐phase (200 mg/cm^3^, right side) phantom. (*B*) Dual‐energy X‐ray absorptiometry (DXA) semi‐automated analysis using the Hologic scanner software. Each vertebra was manually defined as extending from the intervertebral disk to the facets. Phantom calibration of the DXA scanner was performed daily before imaging animals.

All animals underwent a lumbar CT scan in a Siemens Somatom Definition AS 64‐slice scanner (Siemens Healthineers, Munich, Germany) at each of the described five time points. Animals were placed in the scanner in dorsal recumbency under general anesthesia and a Siemens Osteo phantom (Siemens Healthcare, Erlangen, Germany) was placed along the dorsal aspect of the animal's lumbar region to ensure inclusion of bone‐like and water‐like phase reference values in each scan. A single 10‐mm‐thickness slice with a voxel size of 0.32 × 0.32 mm (voltage 80 kVp, current 300 mA) was acquired in the transverse plane of the midsection of three individual lumbar vertebrae, L_3_ through L_5_. Scans were analyzed using the syngo Osteo software (Siemens AG, version VA48A, Munich, Germany). Automatic contour tracing of each vertebral body and the phantom was performed, allowing for automatic separation between trabecular and cortical bone (Fig. 1*A*). The automated segmentation was verified and manual adjustments were performed if the cortical and trabecular bone were misregistered. Mean trabecular volumetric BMD (Tb.vBMD) and mean cortical BMD (Ct.vBMD) were reported for each scanned vertebrae in mgCa‐HA/cm^3^.

### DXA measurements

Immediately after QCT scanning, all animals underwent a DXA scan with a pixel size of 0.90 × 0.90 mm using a Hologic Discovery A scanner (Hologic, Inc., version 13.3.0.1, Marlborough, MA, USA). Animals were positioned in dorsal recumbency on the DXA table and a scan of the lumbar spine region (L_3_ to L_5_) was performed. Scans were performed in triplicate for each animal to ensure minimal disruption of BMD measurements due to positioning. DXA device calibration was carried out using a Hologic spine phantom (Hologic, Inc.) before each scanning time point according to the manufacturer's protocol. Areal BMD (aBMD) of the lumbar vertebrae was determined by manually defining the area between the caudal intervertebral disk to the cranial facets of the vertebral body and calculating the aBMD using the Hologic software (Fig. 1 *B* **)**. aBMD was reported in g/cm^2^ for each vertebra.

### Micro‐computed tomography

The iliac crest biopsy collected from each animal at each time point was used for microCT analysis to quantify the trabecular microarchitecture changes over time. Samples were scanned at a resolution of 10 μm^3^ at 70 kVp, 113 μA, and 500 ms integration time (Scanco μCT 80, version 1.1.15.0, Scanco USA, Inc., Wayne, PA, USA). One region of interest (ROI) (5 mm diameter, 400 slices) was drawn per sample to include only trabecular bone and reconstructed using fixed optimal threshold values (upper bound = 2760.5 HU, lower bound = 456.7 HU). Threshold bounding was confirmed by visual inspection. The following output measures of trabecular microarchitecture were quantified from the three‐dimensional reconstruction of each ROI cylinder: bone volume fraction (BV/TV), trabecular thickness (Tb.Th), trabecular number (Tb.N), and trabecular spacing (Tb.Sp).

### Statistical analysis

BMD measurements and percentage change in BMD were separately assessed for statistical differences at all time points compared with baseline values using a two‐way analysis of variance (ANOVA) with Dunnett's multiple comparisons. MicroCT measurements were assessed for statistical differences at all time points compared with baseline values using a one‐way ANOVA with Dunnett's multiple comparisons. Comparisons among DXA, QCT, and microCT outcomes were made using a Pearson correlation, and a correlation coefficient (*R* ^2^) and 95% confidence interval (CI) was reported for each relationship. An *R* ^2^ value of 0.0–0.19 indicated a very weak correlation, 0.20–0.39 a weak correlation, 0.40–0.59 a moderate correlation, 0.60–0.79 a strong correlation, and 0.80–1.0 a very strong correlation.^(^ ^43^, ^44^ ^)^ Correlation coefficients for aBMD versus combined vBMD (average of Ct.vBMD and Tb.vBMD) were calculated separately for each time point and include values for each lumbar vertebra (L_3_, L_4_, L_5_). When comparing with the iliac crest microCT outcomes, vBMD and aBMD values were averaged across the whole measured lumbar spine (L_3_ to L_5_). The best‐fit line of the relationship was determined by simple linear regression. For all statistical analyses, an alpha value of 0.05 or less (*p* ≤ 0.05) was considered significant (GraphPad Prism 9.5.0, San Diego, CA, USA).

## Results

### DXA measurements

**Fig. 2:**
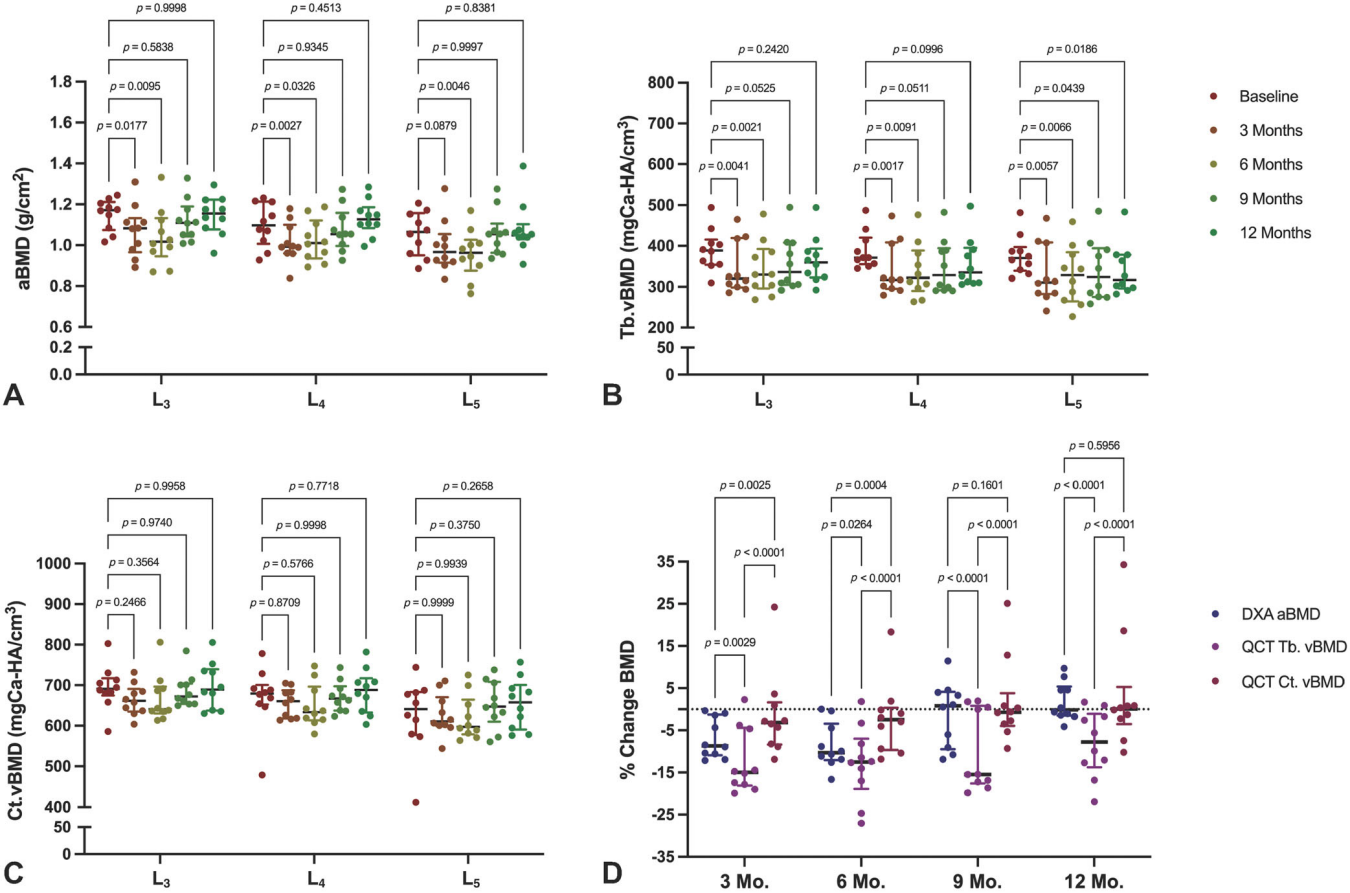
Bone mineral density (BMD) changes across each lumbar vertebrae (L_3_, L_4_, L_5_) in the sheep spine at baseline, 3, 6, 9, and 12 months post‐ovariectomy (OVX) (*N* = 10 subjects). Data are represented as median ± interquartile range with individual animals marked and *p* values provided for individual comparisons. (*A*) Dual‐energy X‐ray absorptiometry (DXA) areal BMD (aBMD) at each time point compared with baseline values. (*B*) Quantitative computed tomography (QCT) trabecular volumetric BMD (Tb.vBMD) values at each time point compared with baseline values. (*C*) QCT cortical vBMD (Ct.vBMD) values at each time point compared with baseline values. (*D*) Percent change of DXA aBMD, QCT Tb.vBMD, and QCT Ct.vBMD averaged across the whole measured spine (L_3_ to L_5_) at each time point compared with baseline values. Statistical comparisons made using a two‐way ANOVA with Dunnett's multiple comparisons.

Baseline DXA aBMD measurements ranged from 0.882 g/cm^2^ to 1.206 g/cm^2^ across all measured vertebrae. Significant decreases in average aBMD relative to baseline were noted across L_3_ and L_4_ at 3 months (*p* = 0.02; *p* = 0.003, respectively) and 6 months (*p* = 0.01; *p* = 0.03, respectively), as well as L_5_ at 6 months (*p* = 0.005) (Fig. 2*A*). There was no change compared with baseline L_3_ or L_4_ aBMD values at 9 and 12 months, and no change to L_5_ values at 3, 9, and 12 months. On average, DXA aBMD measurements decreased by 6.95% (± 4.71%) at 3 months, 8.65% (± 5.40%) at 6 months, 1.28% (± 7.74%) at 9 months, and increased by 1.80% (± 4.56%) at 12 months post‐OVX (Fig. 2*D*) across the whole measured lumbar spine (L_3_ to L_5_) when compared with baseline values.

### QCT measurements

Tb.vBMD measurements ranged from 309.9 mgCa‐HA/cm^3^ to 494.2 mgCa‐HA/cm^3^ at baseline, whereas Ct.vBMD ranged from 478.6 mgCa‐HA/cm^3^ to 802.7 mgCa‐HA/cm^3^. Similar to DXA, average Tb.vBMD significantly decreased when compared with baseline values across L_3_, L_4_, and L_5_ at 3 months (*p* = 0.004; *p* = 0.002; *p* = 0.006, respectively) and 6 months (*p* = 0.002; *p* = 0.009; *p* = 0.007, respectively) (Fig. 2*B*). However, L_5_ Tb.vBMD was also noted to be significantly decreased from baseline at the 9‐month (*p* = 0.04) and 12‐month (*p* = 0.02) time points (Fig. 2*B*). There was no change compared with baseline L_3_ and L_4_ Tb.vBMD values at 9 and 12 months. Average Ct.vBMD did not significantly change between any time points at any of the measured vertebrae (Fig. 2*C*) when compared with baseline values. QCT Tb.vBMD measurements decreased, on average, by 12.47% (±7.61%) at 3 months, 12.90% (±8.76%) at 6 months, 9.93% (± 9.55%) at 9 months, and 8.08% (±7.88%) at 12 months (Fig. 2*D*) post‐OVX across the whole measured lumbar spine (L_3_ to L_5_). Average Ct.vBMD decreased by 1.34% (±10.08%) at 3 months, 2.15% (±8.56%) at 6 months, and increased by 1.68% (±10.02%) at 9 months and 3.35% (±13.23%) at 12 months compared with baseline (Fig. 2*D*).

### Comparison of in‐life imaging modalities

**Fig. 3:**
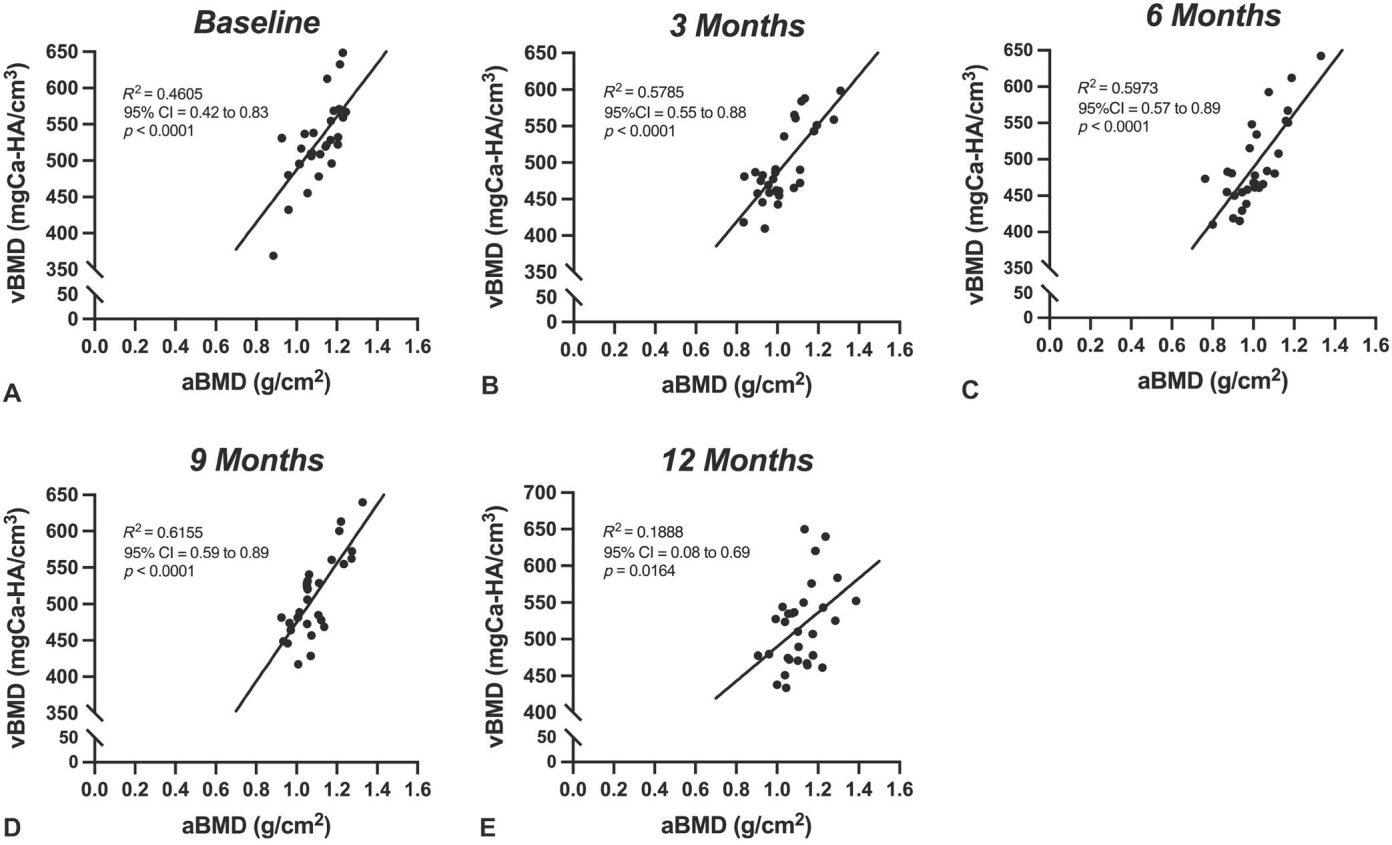
Relationship between dual‐energy X‐ray absorptiometry (DXA) areal bone mineral density (aBMD) and combined quantitative computed tomography (QCT) volumetric BMD (vBMD) (trabecular and cortical vBMD average) of all measured lumbar vertebrae (L_3_, L_4_, and L_5_) (*N* = 10 subjects): (*A*) Correlation between aBMD and vBMD at baseline, before ovariectomy (OVX). (*B*) Correlation between aBMD and vBMD at 3 months after OVX. (*C*) Correlation between aBMD and vBMD at 6 months after OVX. (*D*) Correlation between aBMD and vBMD at 9 months after OVX. (*E*) Correlation between aBMD and vBMD at 12 months after OVX. Statistical comparisons made using a Pearson's correlation and simple linear regression.

Tb.vBMD (QCT) bone loss was significantly greater than both aBMD (DXA) and Ct.vBMD (QCT) at 3 months (*p* = 0.003; *p* < 0.0001, respectively), 6 months (*p* = 0.03; *p* < 0.0001, respectively), 9 months (*p* < 0.0001; *p* < 0.0001, respectively), and 12 months (*p* < 0.0001; *p* < 0.0001, respectively) (Fig. 2*D*). Although aBMD bone loss was also significantly greater than Ct.vBMD at 3 months (*p* = 0.003) and 6 months (*p* = 0.0004), there were no significant differences observed at 9 months (*p* = 0.16) and 12 months (*p* = 0.60) (Fig. 2*D*). When comparing DXA aBMD and combined QCT vBMD (average of Tb. and Ct. vBMD measurements) of L_3_, L_4_, and L_5_ at each of the time points (baseline, 3, 6, 9, and 12 months), the correlation coefficients (*R* ^2^) were 0.46 (moderate, *p* < 0.0001), 0.58 (moderate, *p* < 0.0001), 0.60 (moderate, *p* < 0.0001), 0.62 (strong, *p* < 0.0001), and 0.19 (very weak, *p* = 0.02), respectively (Fig. 3*A–E*).

### Comparison with microCT

**Fig. 4:**
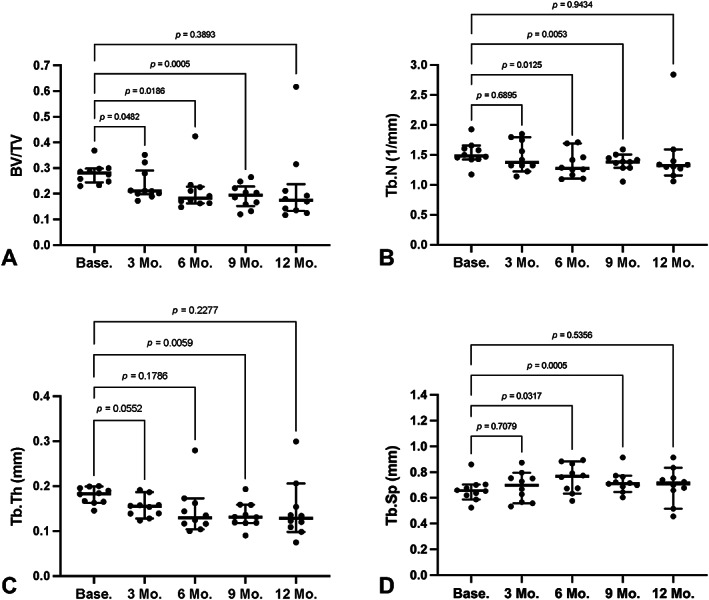
Bone microarchitectural changes measured using microCT in the sheep iliac crest biopsies at baseline, 3, 6, 9, and 12 months post‐ovariectomy (OVX) (*N* = 10 subjects). Data are represented as median ± interquartile range with individual animals marked and *p* values provided for individual comparisons. (*A*) Bone volume fraction (BV/TV) at each time point compared with baseline values. (*B*) Trabecular number (Tb.N) values at each time point compared with baseline values. (*C*) Trabecular thickness (Tb.Th) values at each time point compared with baseline values. (*D*) Trabecular spacing (Tb.Sp) values at each time point compared with baseline values. Statistical comparisons made using a one‐way ANOVA with Dunnett's multiple comparisons.

MicroCT BV/TV of iliac crest trabecular bone decreased significantly at 3 months (*p* = 0.05), 6 months (*p* = 0.02), and 9 months (*p* = 0.0005) compared with baseline (Fig. 4*A*). Iliac crest Tb.N decreased significantly at 6 months (*p* = 0.01) and 9 months (*p* = 0.005) (Fig. 4*B*). Tb.Th decreased significantly at 9 months (*p* = 0.006) (Fig. 4*C*), and Tb.Sp increased significantly at 6 months (*p* = 0.03) and 9 months (*p* = 0.0005) (Fig. 4*D*). Significant moderate correlations were found between lumbar spine Tb.vBMD (QCT) and iliac crest BV/TV (microCT) at baseline (*R* ^2^ = 0.54, *p* = 0.02), 3 months (*R* ^2^ = 0.57, *p* = 0.01), 9 months (*R* ^2^ = 0.56, *p* = 0.01), and 12 months (*R* ^2^ = 0.48, *p* = 0.03), and a significant strong correlation was found at 6 months (*R* ^2^ = 0.66, *p* = 0.004) (Table 1). When comparing lumbar spine aBMD (DXA) and iliac crest BV/TV, significant correlations were only found at 3 months (*R* ^2^ = 0.55, *p* = 0.01) and 6 months (*R* ^2^ = 0.41, *p* = 0.05) (Table 1). Correlations between lumbar spine Tb.vBMD and iliac crest Tb.N (micoCT) were significant at baseline (*R* ^2^ = 0.47, *p* = 0.03), 3 months (*R* ^2^ = 0.57, *p* = 0.01), 9 months (*R* ^2^ = 0.46, *p* = 0.03), and 12 months (*R* ^2^ = 0.62, *p* = 0.007) (Table 1), whereas a significant correlation was only found at 3 months (*R* ^2^ = 0.52, *p* = 0.02) when comparing lumbar spine aBMD and iliac crest Tb.N (Table. 1).

**Table 1:** Pearson Correlations for Relationships Between Iliac Crest Trabecular Bone MicroCT Volume and Microarchitecture Parameters and Bone Mineral Density Acquired by DXA and QCT for 10 Osteoporotic Sheep

MicroCT outcome	Time Point	DXA aBMD (g/cm^2^)		QCT Tb.vBMD (mgCa‐HA/cm^3^)	
		Correlation coefficient (*R* ^2^)	95% confidence interval	Correlation coefficient (*R* ^2^)	95% confidence interval
BV/TV	Baseline	0.2281	−0.22 to 0.85	0.5364*	0.19 to 0.93
	3 months	0.5530*	0.21 to 0.94	0.5661*	0.23 to 0.94
	6 months	0.4057*	0.01 to 0.90	0.6576**	0.37 to 0.95
	9 months	0.2027	−0.25 to 0.84	0.5600*	0.22 to 0.94
	12 months	0.1894	−0.27 to 0.84	0.4826*	0.12 to 0.92
Tb.N (1/mm)	Baseline	0.1378	−0.34 to 0.81	0.4665*	0.09 to 0.92
	3 months	0.5196*	0.17 to 0.93	0.5748*	0.25 to 0.94
	6 months	0.2487	−0.07 to 0.89	0.3403	−0.19 to 0.86
	9 months	0.2068	−0.25 to 0.84	0.4605*	0.09 to 0.92
	12 months	0.1849	−0.27 to 0.83	0.6155**	0.31 to 0.95
Tb.Th (mm)	Baseline	0.0372	−0.50 to 0.73	0.0806	−0.42 to 0.78
	3 months	0.3738	−0.03 to 0.90	0.3088	−0.11 to 0.88
	6 months	0.2081	−0.24 to 0.84	0.5055*	0.15 to 0.93
	9 months	0.0582	−0.46 to 0.76	0.2374	−0.21 to 0.85
	12 months	0.1572	−0.31 to 0.82	0.3460	−0.07 to 0.89
Tb.Sp (mm)	Baseline	0.0404	−0.74 to 0.49	0.2859	−0.87 to 0.14
	3 months	0.5053*	−0.93 to −0.15	0.5557*	−0.94 to −0.22
	6 months	0.2815	−0.87 to 0.15	0.1401	−0.82 to 0.33
	9 months	0.1453	−0.82 to 0.33	0.3461	−0.89 to 0.07
	12 months	0.1049	−0.79 to 0.38	0.2587	−0.86 to 0.18

Abbreviation: aBMD = areal bone mineral density; BV/TV = bone volume over total volume; Ct.vBMD = cortical volumetric bone mineral density; DXA = dual‐energy X‐ray absorptiometry; microCT = micro‐computed tomography; QCT = quantitative computed tomography; Tb.vBMD = trabecular volumetric bone mineral density; Tb.N = trabecular number; Tb.Sp = trabecular spacing; Tb.Th = trabecular thickness.

* *p* < 0.05.

** *p* < 0.01.

## Discussion

This study measured BMD values utilizing DXA, QCT, and microCT across a 12‐month period in an OVX sheep model of osteoporosis. Results showed QCT measurements and BMD losses more closely matching that of the microCT values compared with DXA, supporting our original hypothesis. The lumbar spine, rich in trabecular bone, offers a key insight into early bone loss, and accurate screening can be indicative of an individual's risk for vertebral fracture. Although microCT is not an option in a clinical setting without the use of bone biopsy, QCT and DXA can be utilized to measure changes in BMD in patients noninvasively. Limitations in current bone scanning modalities, primarily the use of DXA, have implored the search for more precise screening tools in the clinical and preclinical spaces. Although there are advantages to the use of DXA, advances in QCT software present a superior alternative to quantifying trabecular and cortical bone densities and early identification of individuals at high risk for fracture. Although radiation exposure is increased in comparison to DXA, the opportunistic use of CT scans^(^ ^45^, ^46^, ^47^, ^48^, ^49^ ^)^ and low‐dose protocols^(^ ^11^, ^50^ ^)^ can reduce the need for additional scanning time and patient exposure.

Previous clinical studies have shown correlative relationships between DXA and QCT BMD values in the lumbar spine,^(^ ^24^, ^44^, ^51^ ^)^ but there have been no clinical and limited preclinical studies looking at the same subjects over time. Although CT Hounsfield units (HU) and DXA aBMD have been compared in dogs,^(^ ^52^, ^53^ ^)^ ours is the first study to directly compare QCT and DXA in the sheep lumbar spine and to correlate those values to microCT outcomes in the same animals over a long‐term study using a preclinical model of osteoporosis. We show moderate to strong correlations (*R* ^2^ ranging from 0.4–0.6) between QCT vBMD and DXA aBMD values at each time point, indicating that changes in BMD observed in an animal on DXA is reflected by similar trending changes in BMD on QCT when cortical and trabecular bone are analyzed together. This correlation is expected, as both cortical and trabecular bone contribute to DXA aBMD. However, when observing the trabecular bone changes alone using QCT, a higher percentage of bone loss is observed at 9 months and 12 months compared with DXA. We suspect this is due to trabecular regions being more susceptible to rapid BMD and microarchitectural changes than cortical bone, which is a key feature of early‐stage bone loss in peri‐ and postmenopausal osteoporosis.^(^ ^17^ ^)^ It is not unusual to observe a “bounce‐back” of BMD values in sheep after osteoporosis induction,^(^ ^54^, ^55^ ^)^ which we indeed observed in this study when we looked at values determined by DXA alone. However, QCT has contrarily shown sustained trabecular BMD loss in the sheep lumbar spine throughout the duration of the 1‐year study. Although we were unable to harvest serial bone biopsies of the lumbar spine trabecular bone as a direct comparison to DXA and QCT measurements, we were able to obtain iliac crest bone biopsies to compare potentially similar trabecular bone changes in the body at a higher resolution using microCT. We observed a continued decrease in iliac crest trabecular bone microCT values BV/TV and Tb.N at 9 months and 12 months when compared with baseline, demonstrating sustained microarchitectural changes to the trabecular bone of the iliac crest after osteoporosis induction. Thus, we suspect that DXA potentially underestimates the actual change in BMD over time in our preclinical models and may inaccurately represent the amount of sustained loss of trabecular bone.

Differences in scanning resolution also played a key role in the outcomes of this study. MicroCT is one of the highest‐resolution scanning tools available to researchers to investigate changes in bone structure. Unfortunately, microCT is an ex vivo method and cannot be utilized in‐life for humans or large animals. Although microCT can be utilized on bone biopsies from patients, a noninvasive scanning technique more indicative of trabecular bone changes is more preferable to understand patient risk for osteoporotic fractures. HR‐pQCT offers a way to look at bone microarchitecture at a higher resolution than standard in‐life imaging modalities while maintaining relatively low radiation exposure to the patient;^(^ ^56^, ^57^ ^)^ however, its application is limited to the distal tibia and radius because of scanner size limitations. Because trabecular bone microarchitecture heavily factors into the overall bone strength and a disruption to microarchitecture increases fracture risk,^(^ ^15^, ^16^, ^58^, ^59^ ^)^ it is probably more useful to screen an anatomical location with a larger trabecular bone area, such as the lumbar spine. We show stronger and more significant correlations at all time points between microCT BV/TV and QCT Tb.vBMD than with DXA aBMD, as well as significant correlations with Tb.N at a greater number of time points than DXA. Higher imaging resolution of QCT versus DXA likely allowed for more precise segmentation of bone and thus a stronger correlation with high‐resolution iliac crest microCT outcomes. This is in agreement with a previous study conducted by Bodic and colleagues, wherein they observed significant correlative relationships between microCT values (BV/TV and Tb.N) and CT HU measurements in the iliac crest of humans, but observed no significant relationship when compared with DXA BMD measurements in the same samples.^(^ ^60^ ^)^ In this way, we demonstrate that lumbar spine QCT more strongly correlates to iliac crest trabecular bone microarchitectural changes than DXA in our sheep model, thus demonstrating higher sensitivity to multiple properties of osteoporotic bone loss.

This study is not without its limitations. Primarily, we acknowledge that the bone biopsies utilized for microCT and the area used for in vivo scanning are different anatomical locations (iliac crest for microCT versus lumbar spine for DXA and QCT). Although we expect that we would observe similar changes in BMD and trabecular microarchitecture at both anatomical locations, we are unable to confirm that the QCT and DXA findings directly apply to the lumbar spine trabecular microarchitecture. Although some previous studies have shown that there are differences in trabecular bone mass and connectivity^(^ ^61^ ^)^ properties across various anatomical sites of the body, significant correlations in mechanical^(^ ^62^ ^)^ and microarchitectural^(^ ^63^ ^)^ properties between the iliac crest and lumbar spine have been demonstrated. Although this has not been verified directly in sheep, similar trends in bone density loss have been observed over time in previous sheep osteoporosis studies between the lumbar spine and iliac crest.^(^ ^33^ ^)^ Future research would be required to directly compare microarchitectural changes between the iliac crest and lumbar vertebrae trabecular bone in the sheep model of osteoporosis. Additionally, we acknowledge that there is subtle variability in bone microarchitecture across the iliac crest that could have impacted our microCT results, as we were not consistent between animals with respect to exact location of iliac crest biopsy collection site at each time point.^(^ ^61^ ^)^ There are inherent limitations with using conventionally raised sheep in research studies, primarily aging of animals. Although we do our best to control for age based on eruption of incisors, it is difficult to exactly determine age in sheep after reaching skeletal maturity. Therefore, we excluded any animals with not yet erupted or worn‐down incisors that would indicate young or old age, estimating an age between 4 and 6 years and controlling for age effects.^(^ ^39^ ^)^ A limitation to the use of QCT in preclinical research is the use of multiple lumbar CT scans over a longitudinal study, which could add up to high radiation exposure and cost. This is an important factor when considering the use of QCT in human subjects or long‐term animal studies. For reference, animals in our study received an approximate effective radiation dose of 0.06 mSv per single QCT lumbar vertebra scan, whereas DXA radiation exposure typically ranges from 0.022 to 0.047 mSv for a full lumbar spine scan.^(^ ^64^ ^)^ There is typically a higher cost of scanning using CT compared with DXA. Additionally, a trained radiology technician is required to operate a CT scanner, whereas less extensive training is required for DXA operation. However, CT may be more readily available to researchers in a preclinical or veterinary setting than DXA. While anesthesia is required for use of both DXA and CT in a preclinical setting, no differences in time required for scanning and animal positioning were observed between modalities. Lastly, the lack of reference data available for QCT leaves researchers and clinicians alike unable to calculate *T*‐scores, as is available with DXA. Further clinical and preclinical studies using QCT in human patients and animal models with osteoporotic, osteopenic, and normal bone are required to build reference data sets for future use in low bone density screening. Although limitations exist with both methods, the available instrumentation ultimately dictates which scanning modality is to be used in a longitudinal preclinical study.

In conclusion, the results of this study indicate that QCT offers a more precise tool to measure lumbar spine trabecular BMD in a large animal osteoporosis model compared with DXA. Accurate in vivo bone imaging modalities can reduce the number of animals needed for preclinical studies and provide more detailed insight into the progression of hormone or age‐related bone loss that can be translated to humans. This preclinical data adds to the growing body of clinical literature on the use of QCT in lieu of DXA to quantify bone density for early and accurate diagnosis of osteoporosis.

## Author Contributions

**Katie T Bisazza:** Conceptualization; data curation; formal analysis; investigation; methodology; project administration; writing – original draft. **Brad B Nelson:** Conceptualization; data curation; investigation; methodology; writing – review and editing. **Katie J Sikes:** Formal analysis; writing – review and editing. **Lucas Nakamura:** Data curation; methodology. **Jeremiah T Easley:** Conceptualization; funding acquisition; investigation; methodology; resources; supervision; writing – review and editing.

### Peer Review

The peer review history for this article is available at https://www.webofscience.com/api/gateway/wos/peer‐review/10.1002/jbm4.10807.

## Disclosures

The authors have nothing to disclose.

## Data Availability

The data that support the findings of this study are available from the corresponding author upon reasonable request.

## References

[1] Vidal M , Thibodaux RJ , Neira LFV , Messina OD . Osteoporosis: a clinical and pharmacological update. Clin Rheumatol. 2019;38(2):385–395. 30542797 10.1007/s10067-018-4370-1

[2] Burge R , Dawson‐Hughes B , Solomon DH , Wong JB , King A , Tosteson A . Incidence and economic burden of osteoporosis‐related fractures in the United States, 2005‐2025. J Bone Miner Res. 2007;22(3):465–475. 17144789 10.1359/jbmr.061113

[3] Kadri A , Binkley N , Daffner SD , Anderson PA . Fracture in patients with normal bone mineral density: an evaluation of the American Orthopaedic Association's own the bone registry. J Bone Joint Surg Am. 2023;105(2):128–136. 36575157 10.2106/JBJS.22.00012

[4] Randell AG , Nguyen TV , Bhalerao N , Silverman SL , Sambrook PN , Eisman JA . Deterioration in quality of life following hip fracture: a prospective study. Osteoporos Int. 2000;11(5):460–466. 10912850 10.1007/s001980070115

[5] Unnanuntana A , Gladnick BP , Donnelly E , Lane JM . The assessment of fracture risk. J Bone Joint Surg Am. 2010;92(3):743–753. 20194335 10.2106/JBJS.I.00919 PMC2827823

[6] Chou SH , LeBoff MS . Vertebral imaging in the diagnosis of osteoporosis: a clinician's perspective. Curr Osteoporos Rep. 2017;15(6):509–520. 29103097 10.1007/s11914-017-0404-x

[7] Xue S , Zhang Y , Qiao W , et al. An updated reference for calculating bone mineral density T‐scores. J Clin Endocrinol Metab. 2021;106(7):e2613–e2621. 33735391 10.1210/clinem/dgab180

[8] Camacho PM , Petak SM , Binkley N , et al. American Association of Clinical Endocrinologists/American College of Endocrinology Clinical Practice Guidelines for the diagnosis and treatment of postmenopausal Osteoporosis‐2020 update. Endocr Pract. 2020;26(Suppl 1):1–46. 10.4158/GL-2020-0524SUPPL 32427503

[9] Wright NC , Looker AC , Saag KG , et al. The recent prevalence of osteoporosis and low bone mass in the United States based on bone mineral density at the femoral neck or lumbar spine. J Bone Miner Res. 2014;29(11):2520–2526. 24771492 10.1002/jbmr.2269 PMC4757905

[10] Kanis JA , McCloskey EV , Johansson H , Oden A , Melton LJ 3rd , Khaltaev N . A reference standard for the description of osteoporosis. Bone. 2008;42(3):467–475. 18180210 10.1016/j.bone.2007.11.001

[11] Damilakis J , Adams JE , Guglielmi G , Link TM . Radiation exposure in X‐ray‐based imaging techniques used in osteoporosis. Eur Radiol. 2010;20(11):2707–2714. 20559834 10.1007/s00330-010-1845-0 PMC2948153

[12] Sebro R , Ashok SS . A statistical approach regarding the diagnosis of osteoporosis and osteopenia from DXA: are we underdiagnosing osteoporosis? JBMR Plus. 2021;5(2):e10444. 33615110 10.1002/jbm4.10444 PMC7872343

[13] Bolotin HH . Inaccuracies inherent in dual‐energy X‐ray absorptiometry in vivo bone mineral densitometry may flaw osteopenic/osteoporotic interpretations and mislead assessment of antiresorptive therapy effectiveness. Bone. 2001;28(5):548–555. 11344055 10.1016/s8756-3282(01)00423-9

[14] Whitmarsh T , Otake Y , Uemura K , Takao M , Sugano N , Sato Y . A cross‐sectional study on the age‐related cortical and trabecular bone changes at the femoral head in elderly female hip fracture patients. Sci Rep. 2019;9(1):305. 30670734 10.1038/s41598-018-36299-y PMC6343024

[15] Chen H , Zhou X , Fujita H , Onozuka M , Kubo KY . Age‐related changes in trabecular and cortical bone microstructure. Int J Endocrinol. 2013;2013:213234. 23573086 10.1155/2013/213234 PMC3614119

[16] Osterhoff G , Morgan EF , Shefelbine SJ , Karim L , McNamara LM , Augat P . Bone mechanical properties and changes with osteoporosis. Injury. 2016;47(Suppl 2):S11–S20. 10.1016/S0020-1383(16)47003-8 PMC4955555 27338221

[17] Finkelstein JS , Brockwell SE , Mehta V , et al. Bone mineral density changes during the menopause transition in a multiethnic cohort of women. J Clin Endocrinol Metab. 2008;93(3):861–868. 18160467 10.1210/jc.2007-1876 PMC2266953

[18] Riggs BL , Melton LJ 3rd. The prevention and treatment of osteoporosis. N Engl J Med. 1992;327(9):620–627. 1640955 10.1056/NEJM199208273270908

[19] Yu EW , Thomas BJ , Brown JK , Finkelstein JS . Simulated increases in body fat and errors in bone mineral density measurements by DXA and QCT. J Bone Miner Res. 2012;27(1):119–124. 21915902 10.1002/jbmr.506 PMC3864640

[20] Guglielmi G , Floriani I , Torri V , et al. Effect of spinal degenerative changes on volumetric bone mineral density of the central skeleton as measured by quantitative computed tomography. Acta Radiol. 2005;46(3):269–275. 15981723 10.1080/02841850510012661

[21] Smith JA , Vento JA , Spencer RP , Tendler BE . Aortic calcification contributing to bone densitometry measurement. J Clin Densitom. 1999;2(2):181–183. 10499978 10.1385/jcd:2:2:181

[22] Liu G , Peacock M , Eilam O , Dorulla G , Braunstein E , Johnston CC . Effect of osteoarthritis in the lumbar spine and hip on bone mineral density and diagnosis of osteoporosis in elderly men and women. Osteoporos Int. 1997;7(6):564–569. 9604053 10.1007/BF02652563

[23] Adams JE . Quantitative computed tomography. Eur J Radiol. 2009;71(3):415–424. 19682815 10.1016/j.ejrad.2009.04.074

[24] Pinto EM , Neves JR , Teixeira A , et al. Efficacy of Hounsfield units measured by lumbar computer tomography on bone density assessment: a systematic review. Spine (Phila Pa 1976). 2022;47(9):702–710. 34468433 10.1097/BRS.0000000000004211

[25] Oei L , Koromani F , Rivadeneira F , Zillikens MC , Oei EH . Quantitative imaging methods in osteoporosis. Quant Imaging Med Surg. 2016;6(6):680–698. 28090446 10.21037/qims.2016.12.13 PMC5219969

[26] Link TM . Osteoporosis imaging: state of the art and advanced imaging. Radiology. 2012;263(1):3–17. 22438439 10.1148/radiol.12110462 PMC3309802

[27] Genant HK , Engelke K , Prevrhal S . Advanced CT bone imaging in osteoporosis. Rheumatology (Oxford). 2008;47(Suppl 4):iv9–iv16. 18556648 10.1093/rheumatology/ken180 PMC2427166

[28] Li N , Li XM , Xu L , Sun WJ , Cheng XG , Tian W . Comparison of QCT and DXA: osteoporosis detection rates in postmenopausal women. Int J Endocrinol. 2013;2013:895474. 23606843 10.1155/2013/895474 PMC3623474

[29] Choksi P , Jepsen KJ , Clines GA . The challenges of diagnosing osteoporosis and the limitations of currently available tools. Clin Diabetes Endocrinol. 2018;4:12. 29862042 10.1186/s40842-018-0062-7 PMC5975657

[30] Sartoretto SC , Uzeda MJ , Miguel FB , Nascimento JR , Ascoli F , Calasans‐Maia MD . Sheep as an experimental model for biomaterial implant evaluation. Acta Ortop Bras. 2016;24(5):262–266. 28149193 10.1590/1413-785220162405161949 PMC5266658

[31] Oheim R , Amling M , Ignatius A , Pogoda P . Large animal model for osteoporosis in humans: the ewe. Eur Cell Mater. 2012;24:372–385. 23147526 10.22203/ecm.v024a27

[32] Turner AS . The sheep as a model for osteoporosis in humans. Vet J. 2002;163(3):232–239. 12090765 10.1053/tvjl.2001.0642

[33] Zarrinkalam MR , Beard H , Schultz CG , Moore RJ . Validation of the sheep as a large animal model for the study of vertebral osteoporosis. Eur Spine J. 2009;18(2):244–253. 19015899 10.1007/s00586-008-0813-8 PMC2899341

[34] Biehl C , Schmitt J , Stoetzel S , et al. DXA reference values of the humanoid sheep model in preclinical studies. PeerJ. 2021;9:e11183. 33986984 10.7717/peerj.11183 PMC8092102

[35] Turner AS , Mallinckrodt CH , Alvis MR , Bryant HU . Dual‐energy X‐ray absorptiometry in sheep: experiences with in vivo and ex vivo studies. Bone. 1995;17(4 Suppl):381S–387S. 8579941 10.1016/8756-3282(95)00315-5

[36] Egermann M , Gerhardt C , Barth A , Maestroni GJ , Schneider E , Alini M . Pinealectomy affects bone mineral density and structure—an experimental study in sheep. BMC Musculoskelet Disord. 2011;12:271. 22115044 10.1186/1471-2474-12-271 PMC3270003

[37] Egermann M , Goldhahn J , Holz R , Schneider E , Lill CA . A sheep model for fracture treatment in osteoporosis: benefits of the model versus animal welfare. Lab Anim. 2008;42(4):453–464. 18782823 10.1258/la.2007.007001

[38] Sigrist IM , Gerhardt C , Alini M , Schneider E , Egermann M . The long‐term effects of ovariectomy on bone metabolism in sheep. J Bone Miner Metab. 2007;25(1):28–35. 17187191 10.1007/s00774-006-0724-x

[39] Cocquyt G , Driessen B , Simoens P . Variability in the eruption of the permanent incisor teeth in sheep. Vet Rec. 2005;157(20):619–623. 16284330 10.1136/vr.157.20.619

[40] James AW , Shen J , Zhang X , et al. NELL‐1 in the treatment of osteoporotic bone loss. Nat Commun. 2015;6:7362. 26082355 10.1038/ncomms8362 PMC4557288

[41] Easley JT , Garofolo SQ , Ruehlman D , Hackett ES . A 3‐portal laparoscopic ovariectomy technique in ewes. Small Rumin Res. 2014;121(2):336–339.

[42] Klopfenstein Bregger MD , Schawalder P , Rahn B , Eckhardt C , Schneider E , Lill C . Optimization of corticosteroid induced osteoporosis in ovariectomized sheep. A bone histomorphometric study. Vet Comp Orthop Traumatol. 2007;20(1):18–23. 17364091

[43] Lucas K , Behrens BA , Nolte I , et al. Comparative investigation of bone mineral density using CT and DEXA in a canine femoral model. J Orthop Res. 2017;35(12):2667–2672. 28387962 10.1002/jor.23574

[44] Alawi M , Begum A , Harraz M , et al. Dual‐energy X‐ray absorptiometry (DEXA) scan versus computed tomography for bone density assessment. Cureus. 2021;13(2):e13261. 33717764 10.7759/cureus.13261 PMC7954087

[45] Lenchik L , Weaver AA , Ward RJ , Boone JM , Boutin RD . Opportunistic screening for osteoporosis using computed tomography: state of the art and argument for paradigm shift. Curr Rheumatol Rep. 2018;20(12):74. 30317448 10.1007/s11926-018-0784-7 PMC7092507

[46] Gausden EB , Nwachukwu BU , Schreiber JJ , Lorich DG , Lane JM . Opportunistic use of CT imaging for osteoporosis screening and bone density assessment: a qualitative systematic review. J Bone Joint Surg Am. 2017;99(18):1580–1590. 28926388 10.2106/JBJS.16.00749

[47] Berger‐Groch J , Thiesen DM , Ntalos D , Hennes F , Hartel MJ . Assessment of bone quality at the lumbar and sacral spine using CT scans: a retrospective feasibility study in 50 comparing CT and DXA data. Eur Spine J. 2020;29(5):1098–1104. 31955257 10.1007/s00586-020-06292-z

[48] Leonhardt Y , May P , Gordijenko O , et al. Opportunistic QCT bone mineral density measurements predicting osteoporotic fractures: a use case in a prospective clinical cohort. Front Endocrinol (Lausanne). 2020;11:586352. 33240220 10.3389/fendo.2020.586352 PMC7680958

[49] Kim KJ , Kim DH , Lee JI , Choi BK , Han IH , Nam KH . Hounsfield units on lumbar computed tomography for predicting regional bone mineral density. Open Med (Wars). 2019;14:545–551. 31410366 10.1515/med-2019-0061 PMC6689205

[50] Goo HW . CT radiation dose optimization and estimation: an update for radiologists. Korean J Radiol. 2012;13(1):1–11. 22247630 10.3348/kjr.2012.13.1.1 PMC3253393

[51] Schreiber JJ , Anderson PA , Rosas HG , Buchholz AL , Au AG . Hounsfield units for assessing bone mineral density and strength: a tool for osteoporosis management. J Bone Joint Surg Am. 2011;93(11):1057–1063. 21655899 10.2106/JBJS.J.00160

[52] Kwon D , Kim J , Lee H , et al. Quantitative computed tomographic evaluation of bone mineral density in beagle dogs: comparison with dual‐energy x‐ray absorptiometry as a gold standard. J Vet Med Sci. 2018;80(4):620–628. 29415919 10.1292/jvms.17-0428 PMC5938190

[53] Lee D , Lee Y , Choi W , et al. Quantitative CT assessment of bone mineral density in dogs with hyperadrenocorticism. J Vet Sci. 2015;16(4):531–542. 26040613 10.4142/jvs.2015.16.4.531 PMC4701747

[54] Goldhahn J , Jenet A , Schneider E , Lill CA . Slow rebound of cancellous bone after mainly steroid‐induced osteoporosis in ovariectomized sheep. J Orthop Trauma. 2005;19(1):23–28. 15668580 10.1097/00005131-200501000-00005

[55] Zarrinkalam MR , Schultz CG , Parkinson IH , Moore RJ . Osteoporotic characteristics persist in the spine of ovariectomized sheep after withdrawal of corticosteroid administration. J Osteoporos. 2012;2012:182509. 23091772 10.1155/2012/182509 PMC3468144

[56] Doi M , Chiba K , Okazaki N , et al. Bone microstructure in healthy men measured by HR‐pQCT: age‐related changes and their relationships with DXA parameters and biochemical markers. Bone. 2022;154:116252. 34743043 10.1016/j.bone.2021.116252

[57] Macdonald HM , Nishiyama KK , Kang J , Hanley DA , Boyd SK . Age‐related patterns of trabecular and cortical bone loss differ between sexes and skeletal sites: a population‐based HR‐pQCT study. J Bone Miner Res. 2011;26(1):50–62. 20593413 10.1002/jbmr.171

[58] Xie F , Zhou B , Wang J , et al. Microstructural properties of trabecular bone autografts: comparison of men and women with and without osteoporosis. Arch Osteoporos. 2018;13(1):18. 29508160 10.1007/s11657-018-0422-z

[59] Parfitt AM , Mathews CH , Villanueva AR , Kleerekoper M , Frame B , Rao DS . Relationships between surface, volume, and thickness of iliac trabecular bone in aging and in osteoporosis. Implications for the microanatomic and cellular mechanisms of bone loss. J Clin Invest. 1983;72(4):1396–1409. 6630513 10.1172/JCI111096 PMC370424

[60] Bodic F , Amouriq Y , Gayet‐Delacroix M , et al. Relationships between bone mass and micro‐architecture at the mandible and iliac bone in edentulous subjects: a dual X‐ray absorptiometry, computerised tomography and microcomputed tomography study. Gerodontology. 2012;29(2):e585–e594. 21711390 10.1111/j.1741-2358.2011.00527.x

[61] Amling M , Herden S , Posl M , Hahn M , Ritzel H , Delling G . Heterogeneity of the skeleton: comparison of the trabecular microarchitecture of the spine, the iliac crest, the femur, and the calcaneus. J Bone Miner Res. 1996;11(1):36–45. 8770695 10.1002/jbmr.5650110107

[62] Britton JM , Davie MW . Mechanical properties of bone from iliac crest and relationship to L5 vertebral bone. Bone. 1990;11(1):21–28. 2331427 10.1016/8756-3282(90)90067-9

[63] Dempster DW , Ferguson‐Pell MW , Mellish RW , et al. Relationships between bone structure in the iliac crest and bone structure and strength in the lumbar spine. Osteoporos Int. 1993;3(2):90–96. 8453196 10.1007/BF01623379

[64] Shi J , Lee S , Uyeda M , et al. Guidelines for dual energy X‐ray absorptiometry analysis of trabecular bone‐rich regions in mice: improved precision, accuracy, and sensitivity for assessing longitudinal bone changes. Tissue Eng Part C Methods. 2016;22(5):451–463. 26956416 10.1089/ten.tec.2015.0383 PMC4870654

